# Diagnostic error and bias in the department of radiology: a pictorial essay

**DOI:** 10.1186/s13244-023-01521-7

**Published:** 2023-10-02

**Authors:** Li Zhang, Xin Wen, Jian-Wei Li, Xu Jiang, Xian-Feng Yang, Meng Li

**Affiliations:** 1 Department of Diagnostic Radiology, National Cancer Center/National Clinical Research Center for Cancer/Cancer Hospital, Chinese Academy of Medical Sciences and Peking Union Medical College, Beijing, 100021 China; 2 Department of Radiology, Drum Tower Hospital Affiliated to Medical School of Nanjing University, Nanjing, China

**Keywords:** Diagnostic imaging, Perceptual errors, Cognitive errors

## Abstract

Diagnostic imaging is an essential and indispensable part of medical diagnosis and treatment, and diagnostic errors or biases are also common in the department of radiology, sometimes even having a severe impact on the diagnosis and treatment of patients. There are various reasons for diagnostic errors or biases in imaging. In this review, we analyze and summarize the causes of diagnostic imaging errors and biases based on practical cases. We propose solutions for dealing with diagnostic imaging errors and reducing their probability, thereby helping radiologists in their clinical practice.

**Critical relevance statement **Diagnostic errors or bias contribute to most medical errors in the radiology department. Solutions for dealing with diagnostic imaging errors are pivotal for patients.

**Key points**

• Diagnostic errors or bias contribute to most medical errors in radiology department.

• Solutions for dealing with diagnostic imaging errors are pivotal for patients.

• This review summarizes the causes of diagnostic errors and offers solutions to them.

**Graphical Abstract:**

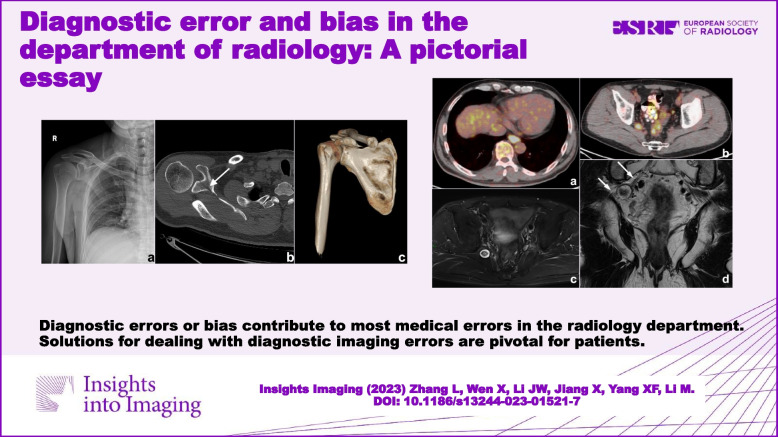

**Supplementary Information:**

The online version contains supplementary material available at 10.1186/s13244-023-01521-7.

## Introduction

Quality assurance is the key component of the modern healthcare system; the structure, processes, and results of clinical work are the major elements of quality management and measurement. Low-quality medical care may result in medical errors, including errors in diagnosis, treatment, prevention, and other types. Medical errors are an important cause of mortality, leading to unnecessary health expenditure [1, 2]. Among them, the percentage of diagnostic errors, including misdiagnoses, missed diagnoses, and delayed diagnoses, is as high as 10–26% of all cases [3, 4]. According to the Committee on Diagnostic Error in Health Care of the Institute of Medicine, diagnostic error is defined as “the failure to (a) establish an accurate and timely explanation of the patient’s health problem(s) or (b) communicate that explanation to the patient” [5]. In addition, medical errors can lead to patient concern, with one study reporting that 38% of patients attending emergency departments had concerns about medical errors, the most common being misdiagnosis (22%) [6]. In the radiology department, most medical errors are classified as diagnostic errors or other errors, such as information failure. Approximately 75% of malpractice lawsuits filed against radiologists relate to diagnostic imaging errors [7].

Diagnostic imaging is the professional interpretation of images; it is a series of uncertain and intricate task processes. The diagnostic process mainly contains 6 steps: assessing the pretest probability of a disease, ensuring the patient’s identity, perception to differentiate negative/positive situations, pattern recognition for the positive findings in radiological diagnosis, differential diagnosis and categorization of the findings, and timely communication of the results in an actionable and reliable format. Therefore, at every step of the process, we are likely to make mistakes due to multiple factors. In addition, the high variability and complexity of imaging techniques and the inherent limitations of the diagnostic capabilities of various imaging modalities also affect the diagnosis. The study of imaging interpretation errors began in the 1940s. At that time, Professor Chamberlain lectured on fluoroscopic errors, including image quality and dark adaptation [8], and presented what may be the earliest traceable study of radiological errors. In 1959, Garland’s research made radiologists aware of the high rate of diagnostic errors in radiology [9]. Subsequent studies have shown that the diagnostic imaging error rate remains, although researchers have performed research and intervention in that area for decades, and the data do not improve compared with Garland’s results. More specifically, if only the number of abnormal imaging results is used as the denominator, the error rate is approximately 30%, but if the number of all imaging results (including abnormal and normal cases) is used as the denominator, the error rate is 3.5–4.5% [10–17]. With the development of modern imaging, although advanced imaging techniques such as computed tomography (CT) and magnetic resonance imaging (MRI) have greatly improved the diagnostic accuracy in the detection of diseases, there are still numerous radiological diagnostic errors due to increased amounts of data and diagnostic information.

These studies suggest that errors in radiology are common and even inevitable. However, to avoid harming the patients primarily, there is a requirement for us to minimize the error rate. This review presents an overview of the common causes and classifications of diagnostic errors in imaging based on our experience and clinical cases. In addition, we also develop some preliminary proposals about how to cope with errors, improve the quality, and help radiologists learn from mistakes. The structure of the error type categorization and the related error management strategies are summarized in Supplementary Table 1.

## Professional causes of diagnostic errors in radiology

The causes of imaging diagnostic errors are complicated and often coexist for multiple reasons. In this article, we mainly discuss two types: perceptual error and cognitive error. A perceptual error can be referred to as a “miss,” which means an important finding is not observed. Similarly, cognitive errors can be considered “misunderstanding,” which means an unusual image is found but subjected to faulty reasoning, or the diagnostic classification of the imaging abnormality is generally correct, but there is inadequate interpretation or complacency due to cognitive bias. Perceptual errors account for approximately 60 to 80% of diagnostic reporting errors, and the proportion of cognitive mistakes is approximately 20 to 40% [10, 18]. Next, we will detail the common causes and examples of these two types of errors based on clinical practice.

### Perceptual errors

Images are the basis of diagnosis, so inappropriate or incomplete scan protocols, image artifacts, and low-quality images caused by limitations of equipment and post-processing software are important objective causes of image reporting errors. In radiology department, quality control focuses on technical performance (artifacts, selection of the study region, etc.) and diagnostic performance (detection of pathology, terminological errors, etc.) [19]. Solutions to these errors include the increasing investment of time and effort in scan and equipment development and routine image quality control, thereby reducing diagnostic errors caused by equipment issues. Apart from that, most perception errors occur when doctors fail to find a meaningful lesion in images (search error), when a lesion is noted for a short time but not given sufficient attention (recognition error), or when doctors attach importance to the lesions but do not provide the correct diagnosis (decision error) [20, 21]. Based on our clinical experience and literature published, we categorize perceptual errors into the following causes.

#### Perceptual errors related to lesion size and density/signal

**Fig. 1:**
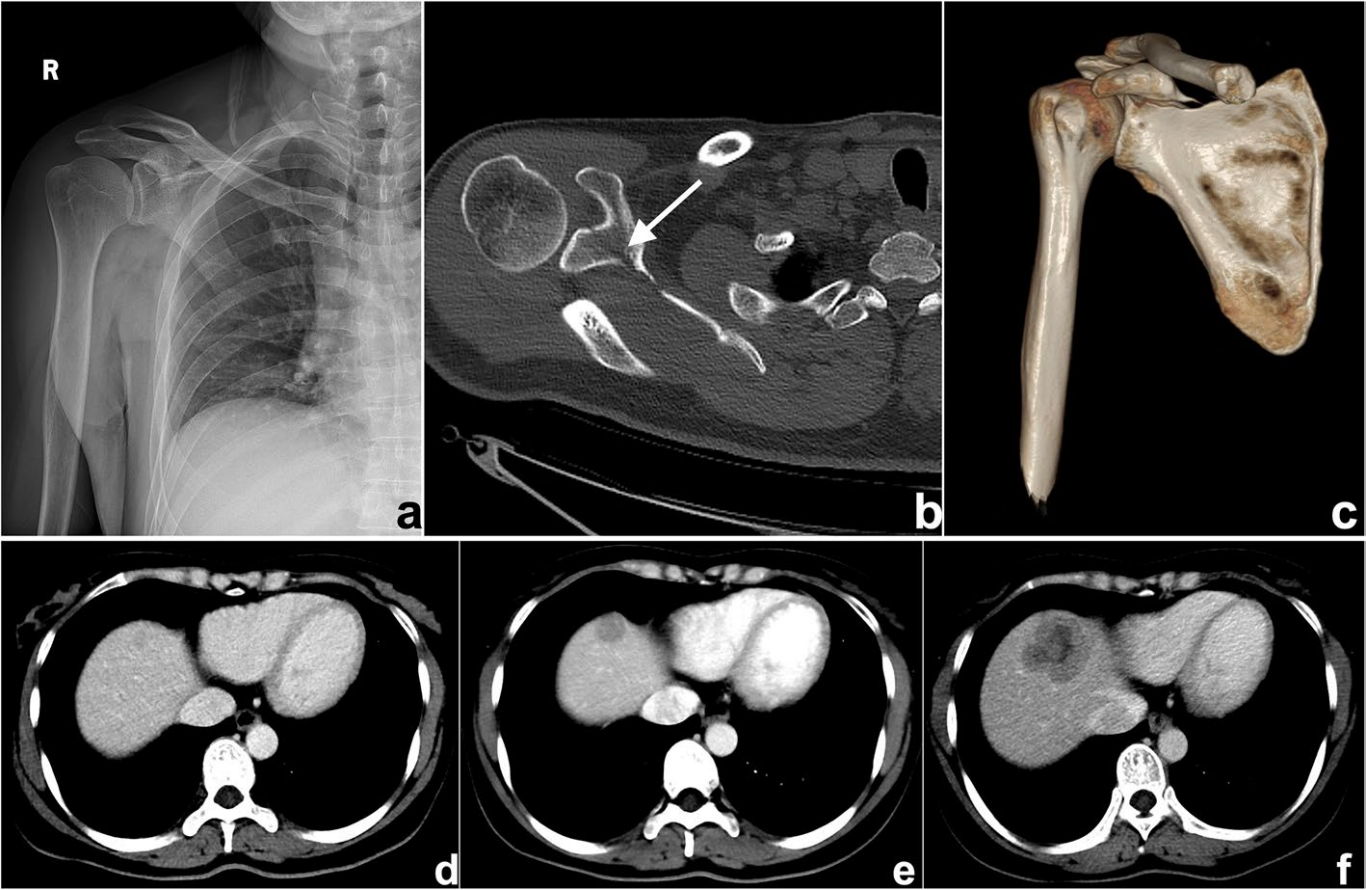
**a**–**c** A 47-year-old man suffering from right shoulder pain for 3 days after trauma. **a** Plain radiography was reported to be normal because the radiologist missed the subtle low-density fracture line due to the overlapping position and careless reading. Axial computed tomography (CT) image (**b**) and three-dimensional (3D) reconstruction (**c**) showed scapular fractures below the coracoid (arrow). **d**–**f** Gradually enlarged low-density liver metastasis in a 32-year-old woman after radical resection and chemotherapy of sigmoid cancer. The images demonstrated an ill-defined margin and heterogeneous enhancement. The lesion was not detected and reported in a timely manner from the first two CT scans (**d**, **e**) because it was small and located at the top of the liver

This is probably one of the most common causes of search errors or missed diagnoses: the lesion is too small to attract the radiologist’s attention. Among such errors, search errors due to small lesions may be the most common; clinically, they include missing small pulmonary nodules, fractures, small liver foci (Fig. 1), and abdominal lymphadenopathy. Moreover, the similarity of the density/signal of the lesion to that of the surrounding tissue also contributes to the omission of lesions. The solutions include increased time for careful reading, bilateral contrast, and postprocessing methods such as maximum intensity projection (MIP), multiplanar reformation (MPR), and three-dimensional (3D) reconstruction.

#### Perceptual errors related to the location/type of lesions

**Fig. 2:**
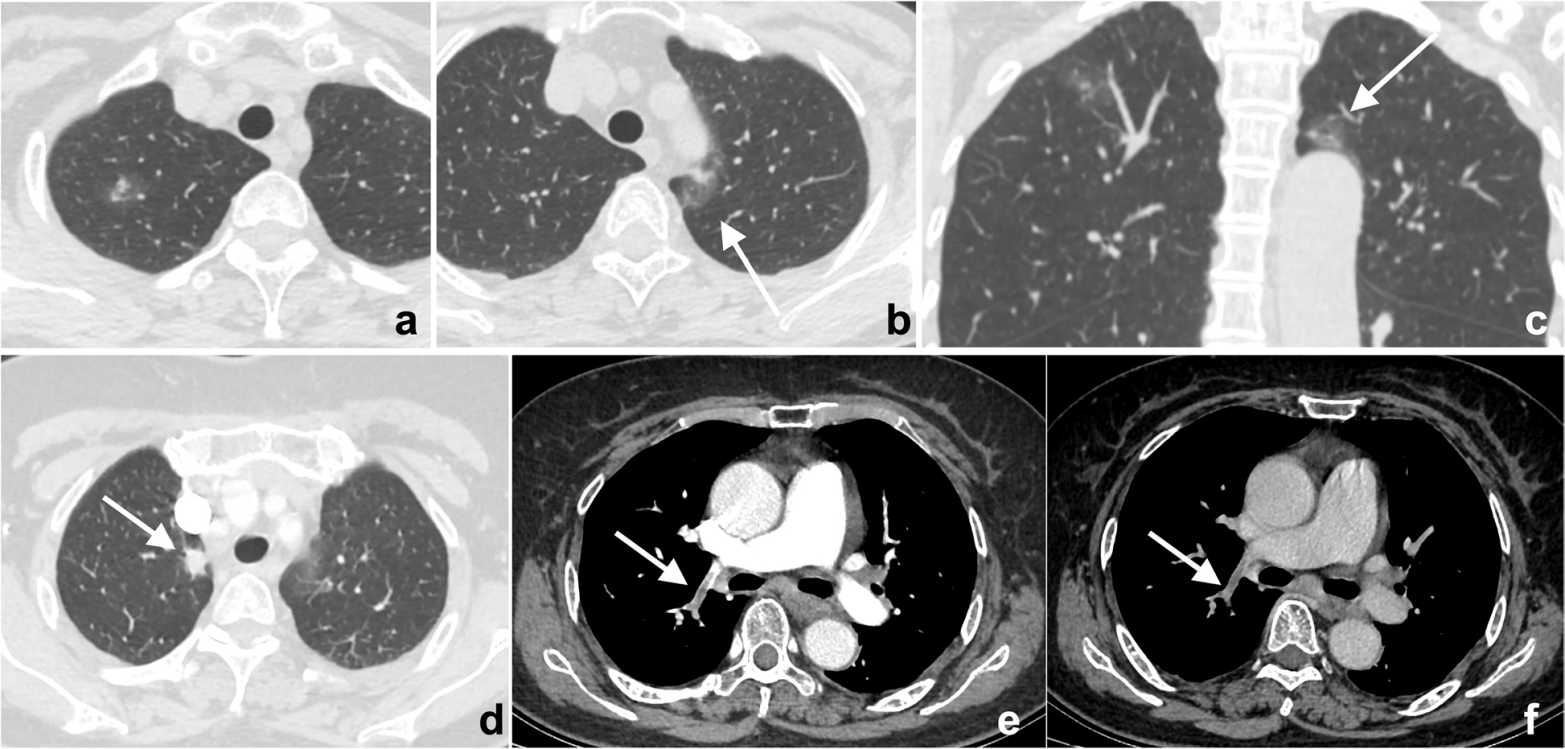
**a**–**c** Double primary lung adenocarcinoma in a 60-year-old woman. **a** Radiologists stopped searching for other lesions after detecting the subsolid nodule in the tip of the right upper lobe, missing another subsolid nodule (arrows) in the posterior apical segment of the left upper lobe, above the aortic arch and next to the mediastinum. Multiplanar reformation (MPR) demonstrated that the nodule (arrow) was above the aortic arch where there was a blind area commonly encountered in image reading. **d**–**f** A 72-year-old woman affected by COVID-19. The enhanced CT presented a description of the nodule (arrows) in the right upper lobe along with the burr sign of lobes, thus prompting the consideration of lung cancer. However, the low-density filling defect in the right upper pulmonary artery, which was a sign of pulmonary artery embolism, was missed

**Fig. 3:**
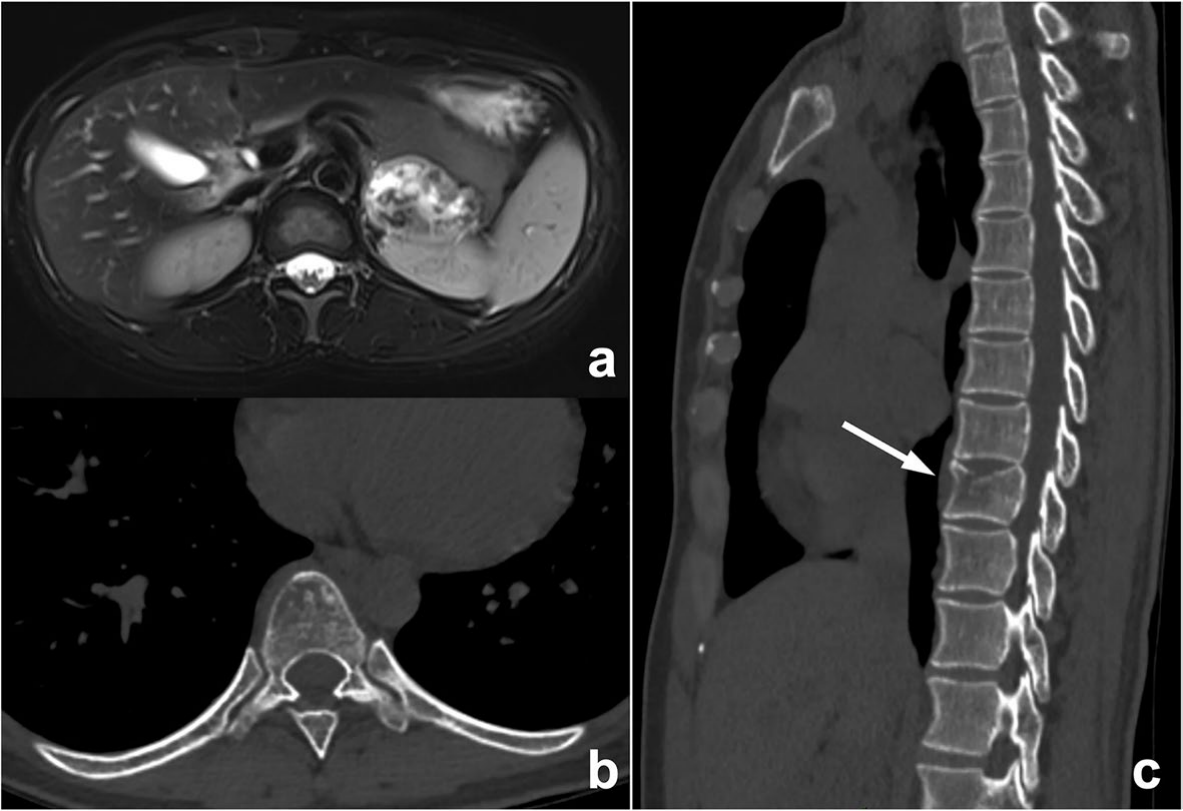
An abnormal signal mass of the left adrenal gland presented by abdominal MRI in a 39-year-old woman. In the routine preoperative chest CT scan (**b**, **c**), bone metastasis (arrow) was missed because radiologists mainly observed the pulmonary and mediastinal window but ignored the bone window. The diagnosis of postoperative pathology was left adrenocortical adenocarcinoma

Whether the lesion is missed is also dependent on its location. If it exists in blind spots or outside the regions of interest (ROIs), such as unique anatomical sites, structural overlaps, the last slice of the scanning field of view (SFOV), the edge of the image, or a corner that is hard to notice, its location may lead to it being missed. This kind of error is known as the location error, and some researchers call it the inattentional bias. For instance, these locations in the chest include the apex of the lung, cardiophrenic angle, parahilar and paraaortic regions, bones, and pulmonary artery. Imaging reading blind spots are a common objective cause of perceptual errors, accounting for 7% of radiological diagnostic errors [10] (Fig. 2). There are some disease entities that can be easily missed. A retrospective study of 122 imaging reports over 2 years demonstrated that the most easily missed types were lymph node metastases of the abdominal and pelvic cavities, bone metastases, malignant lesions of the abdomen and pelvis, fractures, pulmonary nodules, and pulmonary embolism [22] (Fig. 2). This may be due to the high prevalence of these diseases themselves or the fact that these lesions tend to be small or in anatomical blind spots. In addition, bone metastases are often missed because the bone window is not analyzed during body CT reading (Fig. 3). Solutions to this problem include increasing time for comprehensive imaging reading and being more familiar with common blind areas, easily missed lesion types, and the metastatic pattern of neoplastic diseases.

#### Insufficiency of clinical information

**Fig. 4:**
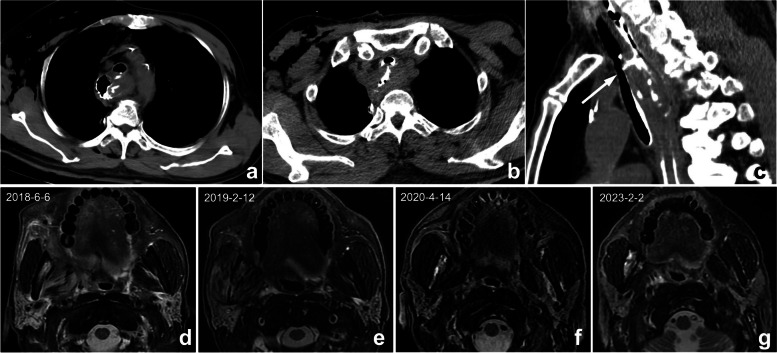
**a**–**c** Postoperative changes in esophageal cancer in a 67-year-old man. **a**, **b** Axial CT showed the postoperative changes in esophageal cancer. However, because the clinical request form did not provide a reminder, radiologists only described postoperative changes and failed to mention tracheoesophageal fistula. Multiplanar reformation (MPR) can better display the fistula (arrow). According to the administration of oral contrast agent, radiologists should understand that the doctor wants to observe if a fistula occurs. (**d**-**g**) A 56-year-old man with right oropharyngeal carcinoma after radiotherapy. Four MRIs at different times showed abnormal signals in the right mandible. The first two reports (**d**, **e**) did not mention abnormal signals, illustrating a perceptual error. In the third exam (**f**), the radiologist found but mistook it as bone metastasis, illustrating a cognitive error. The patient’s frequent swollen gums since 2018 were omitted, and this syndrome improved substantially after anti-inflammatory therapy. The diagnosis should be right mandibular osteoradionecrosis with osteomyelitis, but radiologists misdiagnosed it because they were not familiar with radiotherapy complications and diagnostic bias

A clinically provided request form is often the only way to obtain a history. If the radiologist fails to read the request form carefully or the clinician does not write the information clearly on the request form or update the patient’s clinical status promptly, missed diagnosis or misdiagnosis may ensue. For example, postoperative complications are likely to be missed for postoperative patients because the medical history is usually not updated in time, and radiologists are usually less sensitive to postoperative complications (Fig. 4). Solutions to this problem include improving hospital information systems to ensure adequate clinical and pathological information and increasing the sensitivity of radiologists toward complications following surgery.

#### Perceptual errors related to thinking bias

Some perceptual errors are related to the thinking bias of the human brain, the most common being the satisfaction of search, which refers to decreased vigilance to other abnormalities after detecting the first lesion, resulting in the termination of reading and the omission of other vital lesions (Fig. 2). This is a common cause of errors, contributing approximately 22% [10]. All of these situations have been well documented in the musculoskeletal system [23]. Solutions include reading images systematically, initiating a secondary search to continue looking for others after finding the first abnormality, and having a comprehensive knowledge of common diagnostic combinations.

### Cognitive errors

Cognitive errors have more subjective elements; therefore, they are more complicated. Researchers have most frequently classified the causes of imaging diagnosis or cognitive errors by using the Kim-Mansfield Radiologic Error Classification System [10]. It should be pointed out that careless terminological errors such as reversing left and right writing and misspellings and huge measurement errors are also common reporting errors that are likely to be misunderstood by patients, which may shake patient confidence and lead to unpleasant arguments; in particular, reversing left and right writing errors can sometimes result in severe medical errors and disputes. This type of error is not included in this review.

#### Cognitive errors related to lack of professional knowledge

**Fig. 5:**
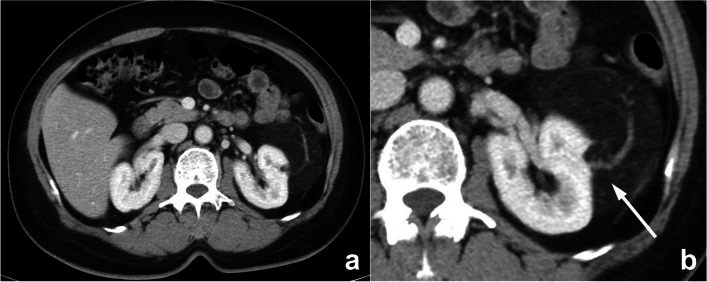
A mass revealed by physical examination in the left kidney of a 58-year-old woman. **a** An enhanced, axial abdominal CT image showed a left pararenal fat-density mass, which was diagnosed as liposarcoma. However, this lesion should have been diagnosed as angiomyolipoma instead of liposarcoma. The misdiagnosis may be due to missing key signs such as large blood vessels (arrow) in the tumor and the renal cortical “split sign”

Errors due to a lack of knowledge often occur among medical students and junior physicians. The insufficiency of experience and expertise can easily lead to the misdiagnosis of lesions. For example, beginners may easily mislocate frontal lobe lesions to the parietal lobe in transverse axial brain CT or MRI images. Alternatively, in chest CT diagnosis, the pericardial recess is easily misdiagnosed as enlarged lymph nodes. Moreover, if pivotal signs are missed, the diagnosis of two similar lesions can also be confused (Fig. 5). Solutions include attaching importance to the training of professional knowledge by report writing training and continuing education for radiologists.

#### Cognitive errors related to the prior examination and report

**Fig. 6:**
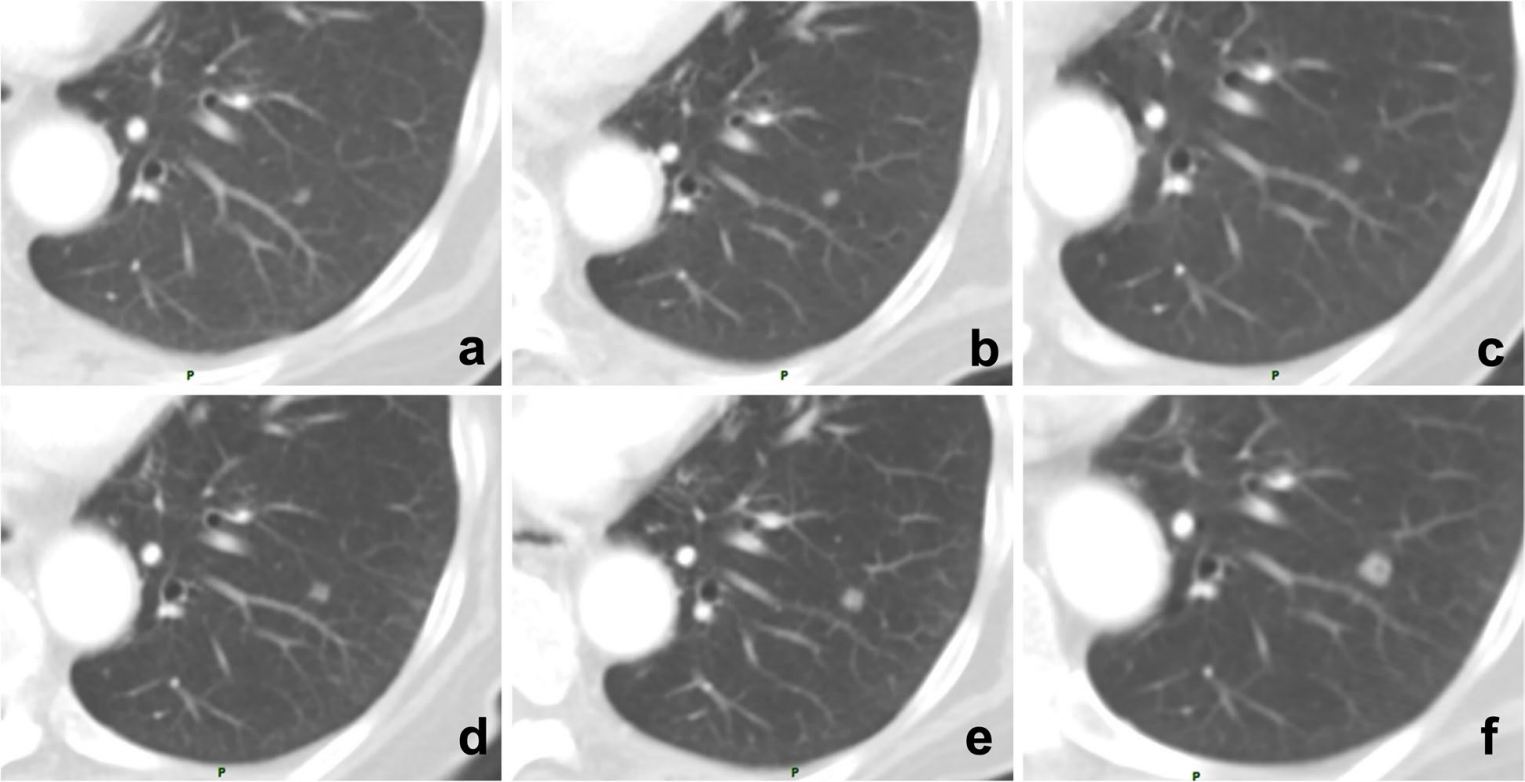
A 71-year-old man underwent multiple follow-up visits after colon cancer surgery. **a**–**f** Images showed a small nodule in the lower lobe of the left lung, but the radiologist only compared the current CT image with that of the last examination and reported no changes. By comparing each CT image, the gradually enlarging nodule was recognized, and follow-up imaging confirmed it as a metastatic tumor

**Fig. 7:**
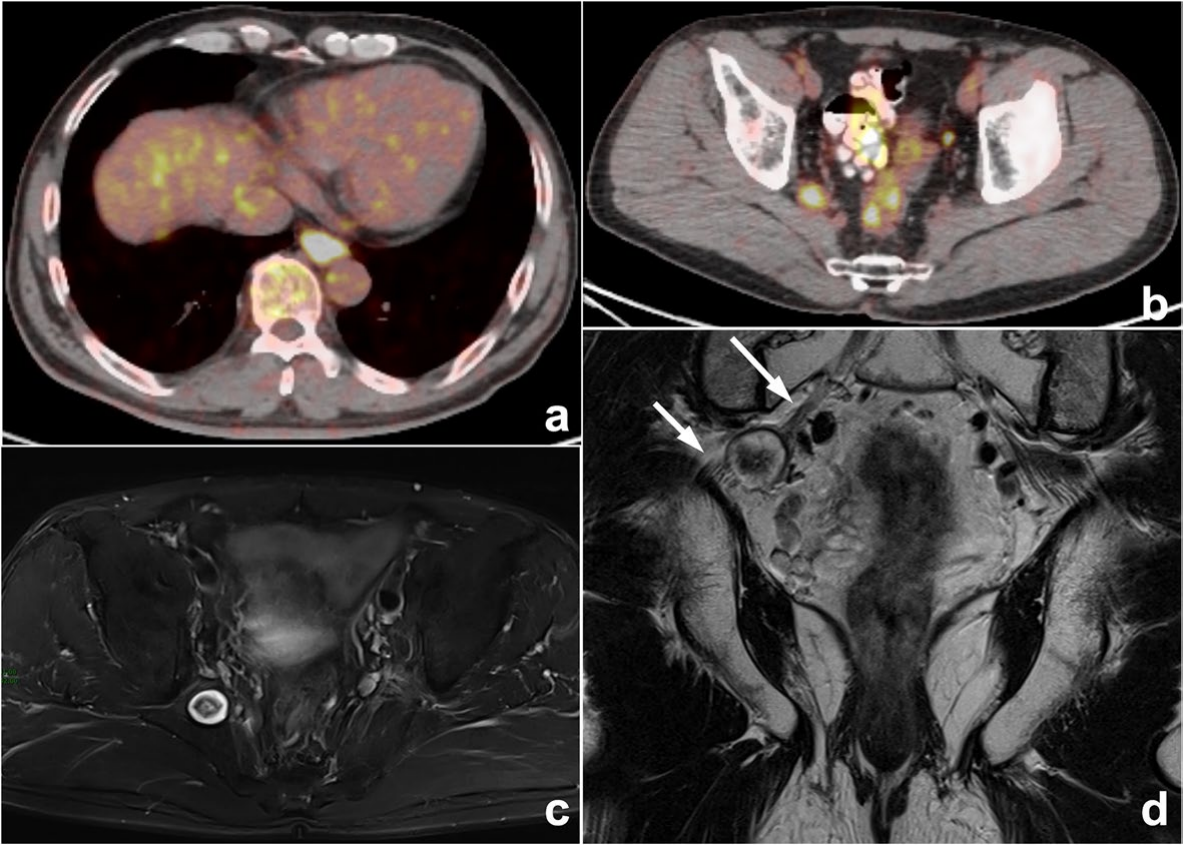
Esophageal carcinoma with multiple systemic metastases reported by PET-CT in a 60-year-old man with dysphagia for 1 month. **a**, **b** PET-CT images showed a mildly elevated pelvic uptake nodule in the right pelvis, which was suspected to be metastasis. **c**, **d** Therefore, the radiologist was influenced by the previous esophageal carcinoma and PET-CT reports, ignoring typical neurogenic tumor signs such as the “target sign” and the close association with the sacral plexus nerve (arrows). Eventually, the right pelvic nodule was misdiagnosed as metastasis in the MRI report. This is a typical example of prior report bias

References to previous reports and comparison of lesions are of great necessity in patients undergoing follow-up or response evaluation. When comparing lesions, it is better to compare them with an earlier or baseline examination rather than just the previous examination; otherwise, the error of comparison will occur (Fig. 6). Moreover, a lack of reference to earlier reports can sometimes lead to misdiagnosis, and excessive trust or reliance on previously reported diagnoses can also lead to diagnostic biases or errors. Some researchers call it prior report bias, alliterative error, or satisfaction of report bias (Fig. 7). Solutions include reading the images carefully and making a diagnosis before reviewing previous reports, revisiting and refining previous reports, and considering a second diagnosis.

#### Cognitive errors related to clinical information

The clinical history is crucial to the diagnosis. Because the request form does not provide a complete or accurate clinical history or is not sensitive enough to specific clinical histories, such as side effects after treatment, radiologists often make false decisions (Fig. 4). Therefore, when the diagnosis is difficult, it is important for radiologists to ask for the clinical history or physical examination in person.

#### Cognitive errors related to thinking cognitive bias

Thinking biases are tendencies of our cognitive functions to obey certain patterns that are not always productive. Various cognitive biases can keep us “locked” on an irrelevant finding, leading to a wrong diagnosis and preventing an objective interpretation. The most frequent and important cognitive biases in our work include anchoring bias, confirmation bias, availability bias, and attribution bias [24].

**Fig. 8:**
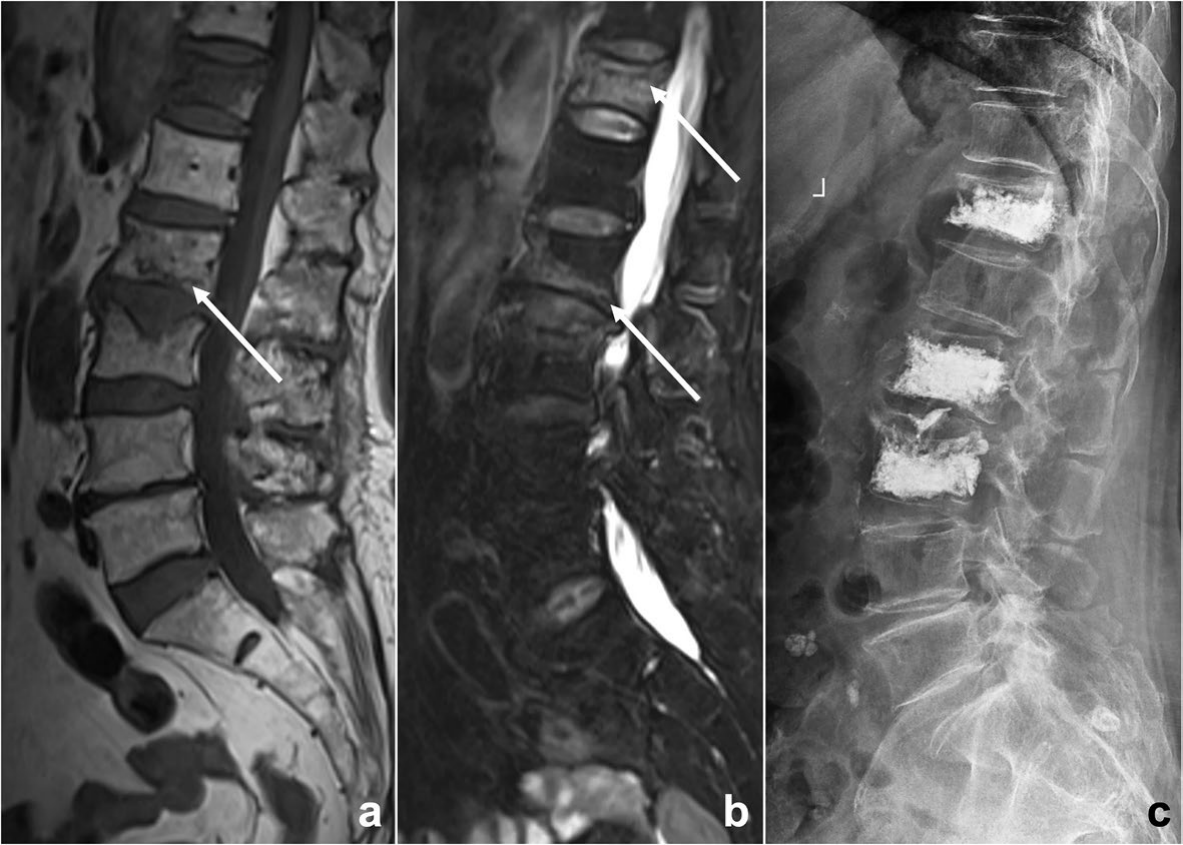
An 81-year-old woman suffered from low back pain for a week after lymphoma chemotherapy. Lumbar MRI T_1_WI (**a**) and T_2_WI/FS (**b**) illustrated multiple abnormal vertebral signals with mild compression fractures. The radiologist misdiagnosed it as a malignant lesion because of the patient’s clinical history of malignant tumor, missing some signs of benign compression fracture such as linear low signal under the endplate, strip-like abnormal signal (arrows), and Schmorl’s nodes. This diagnostic bias is classified as anchoring bias and confirmation bias. After the patient underwent vertebral biopsy and arthroplasty (**c**), no evidence of malignant tumor was found by pathology, and a benign compression fracture was diagnosed

Anchoring bias and confirmation bias can be considered fixed mindset bias. Anchoring bias means that a doctor fixes on his or her initial diagnostic impression too early and ignores subsequently acquired new signs or signs that conflict with the initial impression. Confirmation bias refers to the situation in which when a particular point of view is subjectively supported, we tend to seek information that can help the original point of view but ignore information that may overthrow the original point of view. Regarding the solutions, before the final diagnosis is made, all available evidence should be reviewed and gathered, especially evidence supporting a different opinion, and then we can consider a second diagnosis. For instance, when we find vertebral compression fractures in patients with a history of malignancy, we are likely to consider metastatic tumors, and signs supporting benign diseases may be ignored (Fig. 8).

**Fig. 9:**
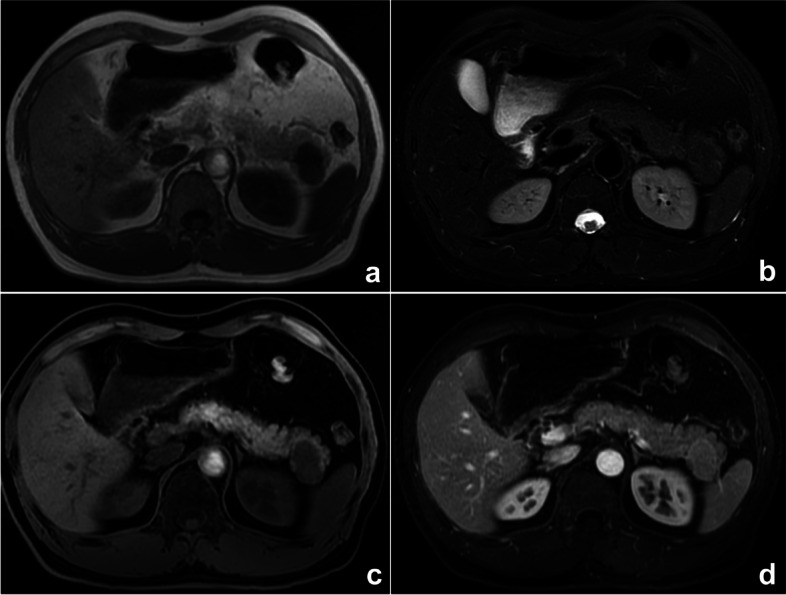
A space-occupying lesion of the pancreatic tail in a 52-year-old man. Axial MRI T_1_WI (**a**), T_2_WI/FS (**b**), T_1_WI/FS (**c**), and enhanced (**d**) sequences all showed that the lesion’s signal was similar to that of the spleen, but our radiologist’s diagnosis was pancreatic neuroendocrine tumor, which was relatively common, rather than a rarely occurring ectopic spleen. Finally, the surgical pathology confirmed an ectopic spleen in the pancreas. This thinking bias is classified as zebra retreat bias

The definition of availability bias is judging the possibility of an event based on how easily and frequently it comes to mind. Another similar cognitive bias is called zebra retreat bias, which refers to a condition in which the patient’s history and imaging findings support a rare diagnosis, but the radiologist is afraid to make the correct diagnosis because of its rarity [25] (Fig. 9). Solutions include using objective data of the disease incidence to correlate with the radiologist’s diagnostic rates and make a differential diagnosis.

**Fig. 10:**
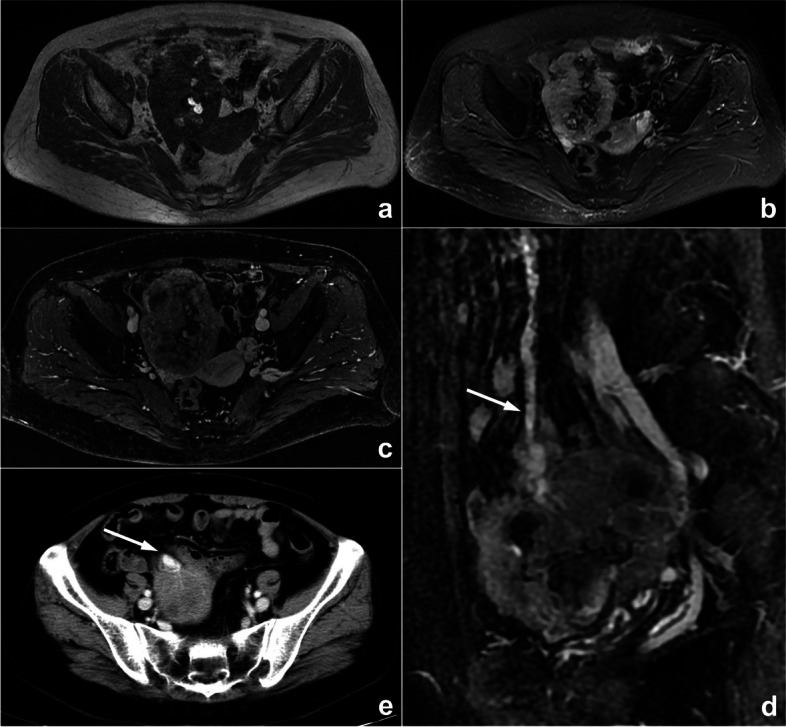
A palpable pelvic mass that was diagnosed as an ovarian tumor by the gynecologist in a 57-year-old woman who presented in gynecological clinics. MRI scan (**a**–**c**) showed a pelvic mass with hemorrhage-like confounding hyperintensity on T_1_WI and hypointensity on T_2_WI/FS internally. An enhanced scan represented heterogeneous enhancement. The mass was closely related to the small intestine, and blood supply by large mesenteric vessels (arrows) could also be observed on enhanced sagittal MRI (**d**) and axial CT (**e**) images, suggesting intestinal original disease. However, the radiologist mistook small intestinal stromal tumors as ovarian tumors because of the gynecologist’s diagnosis. This is classified as attribution bias

Attribution bias means that some specialist doctors are more willing to favor the diagnosis of diseases in their specialty. For radiologists, disease stereotypes are often based on information provided by the department where the patient is seen or the request form. Other analogical biases, such as framing bias, draw different conclusions from the same information because of the different ways or order in which information is presented. For radiologists, misdiagnosis often occurs due to the preconception of clinical information (Fig. 10). Solutions include realizing that initial clinical impressions can sometimes be wrong and reviewing the images before checking the clinical history.

Other cognitive biases include outcome bias and premature closure. The former refers to the tendency of doctors to prefer diagnostic decisions that lead to a better outcome for the patient [25], and the latter refers to regarding a preliminary diagnosis as the premature conclusion [26]. The above errors often occur in multiple combinations (Fig. 4). Solutions include realizing the above cognitive biases that can influence our diagnosis and conducting a complete and accurate diagnosis with a correct attitude.

## Non-professional causes of diagnostic errors in radiology

Many non-professional causes can affect the accuracy of reports during the workflow of radiologists, and fatigue is one of the most important causes [20, 27]. For instance, fatigue from lack of sleep has been identified as a contributing factor in many severe accidents [28, 29]. Some studies have shown that the error rate in diagnosis reports is higher during the night shift, especially after midnight [30]. More importantly, the workload of radiologists has relentlessly increased and is a frequent reason for burnout. Burnout is “a syndrome resulting from chronic workplace stress that has not been successfully managed.” A recent Medscape survey found that 47% of radiologists suffer from burnout [31, 32]. In addition, the rapid reading of images is unavoidable in real work due to the inevitable increase in workload [33]. Under these circumstances, errors the radiologists make will be regarded as reckless readings in the case of medical lawsuits, and these lawsuits will allege that missed diagnoses are due to radiologists spending insufficient time analyzing images. Although the authors of a recent article advocated putting limits on shift hours and workload for radiologists, there is a lack of scientific measures to weigh the workload [34], responsibilities, and reading speed of radiologists.

Another common reason is the inadequate attention of the readers. Currently, radiologists are involved in multidisciplinary teams (MDTs) and scientific teaching meetings in addition to reading images. It has been demonstrated that additional interruptions can lead to a 12% decrease in report correctness [35]. In addition, as a profession requiring intense attention, high visual perception, and cognitive demands, radiologists need optimal physiological conditions. However, approximately 58% of radiologists suffer neck and shoulder pain, back pain, carpal tunnel syndrome, eye strain, headache, and other symptoms that may interfere with work [36, 37]. Solutions to cope with these non-professional factors include taking regular breaks to avoid fatigue, moving and standing from time to time when reading images, minimizing interruptions in the diagnostic process caused by phone calls, using height-adjustable desks and ergonomic chairs, adjusting the brightness of indoor lighting and screens, and reducing ambient noise [38].

## Judgment and systematic strategies for diagnostic errors in radiology

Defining diagnostic errors is a difficult task due to the inherent subjectivity of image interpretation, especially for cognitive errors. First, it is crucial to distinguish between “errors” and observer “discrepancy” when discussing errors in diagnostic radiology. The term “error” implies that there is no potential for argument about what is “correct” and that the reporting radiologist should have been able to make the proper diagnosis or report but was unable to do so. The word “discrepancy” stands for justifiable differences of opinion between colleagues [39, 40]. Second, radiological reports not only accurately convey the presence of abnormalities but also the radiologist’s opinion and the level of diagnostic confidence that expresses a particular level of certainty in the suggested diagnoses [41]. However, because imaging findings are often nonspecific, radiological conclusions usually cannot be clear or definite in clinical work. Sometimes, radiologists will adopt protective measures such as vague or descriptive diagnoses and lengthy differential diagnoses when they want to avoid mistakes and disputes. In general, the interpretation of diagnostic imaging studies relies on consensus expert opinion to determine diagnostic errors [42]. In the process of determining errors in radiology, there are also certain thinking or cognitive biases, such as hindsight bias, which means experts retrospectively downplay or underestimate the difficulties and challenges of the initial diagnosis after the diagnosis of a lesion has been confirmed or additional information has been added, especially if fatality discussions or medical errors/disputes discussions [7, 26, 43, 44].

Previous errors in radiological diagnosis are often attributed to individual carelessness, negligence, or poor performance. Therefore, the solutions for reducing the error rate often focus on improving individual ability and responsibility, as illustrated by earlier descriptions in this article of countermeasures to strengthen the influencing factors. However, there is growing recognition of the limitations of radiologists in image perception and cognition involving a series of complex diagnostic decisions that cause the inevitability of errors. Developing systematic policies and approaches to reduce errors is an effective measure and may involve several approaches, such as multiple reviewer report system, using structured reporting templates and reporting and data system (RADS), error measurement or detection strategies such as electronic trigger tools and checklists to detect “wrong-side” misidentification errors [45], report writing training and continuing education for radiologists, holding radiology quality control meetings and providing peer feedback learning, improving hospital information systems to ensure adequate clinical and pathological information, enhancing communication with doctors or patients, matching workload to staffing, and promoting the application of artificial intelligence (AI).

Multiple reviewer report system can ensure diagnostic accuracy, but they may increase human labor costs. However, in the double-reviewer report system, the reviewers are mainly medical students and junior radiologists; therefore, the application of this system is the most feasible way to balance labor costs and report quality, which also has educational significance for junior reviewers. In difficult cases, we can hold more MDTs with several senior doctors to make correct diagnoses. Moreover, with its rapid development, artificial intelligence (AI) is bound to help doctors reduce missed diagnoses of lesions, improve diagnostic efficiency, and decrease human labor costs. For individual difficult cases, group discussions with wide participation are needed, which can help reduce the diagnostic bias of senior doctors and contribute to residents’ education. For radiology quality control meetings, near misses are potential learning opportunities that should be used to promote quality controls and radiologists’ self-improvement in an attempt to prevent future accidents. In addition, an unbiased “no blame” culture should be introduced and ensured as a method to focus attention on understanding case error instead of individual radiologists and improve the quality of care by learning from mistakes [46, 47].

## Conclusion

In summary, this review presents an analysis of the causes of diagnostic errors in imaging and provides solutions for them with the objective of helping radiologists reduce errors in clinical practice. Quality control of imaging diagnosis is a challenging problem that will need to be addressed by more efforts and research in the future.

### Supplementary Information

**Additional file 1: Supplementary Table 1.** Summarized errors’ types categorization and related errors management strategies.

## Data Availability

Data sharing is not applicable to this article, as no datasets were generated or analyzed during the current study.

## Abbreviations

3D: Three-dimensional
AI: Artificial intelligence
CT: Computed tomography
MDT: Multidisciplinary team
MIP: Maximum intensity projection
MPR: Multiplanar reformation
MRI: Magnetic resonance imaging
RADS: Reporting and data system
ROI: Region of interest
SFOV: Scanning field of view
