# Supplementary material for: Diagnostic error and bias in the department of radiology: a pictorial essay
Source: Insights Imaging. 2023 Oct 2;14:163. doi: 10.1186/s13244-023-01521-7 (PMC10545608; doi:10.1186/s13244-023-01521-7)
Supplement: Supplementary file 1 — Additional file 1: Supplementary Table 1. Summarized errors’ types categorization and related errors management strategies. [file 13244_2023_1521_MOESM1_ESM.docx]

**Diagnostic error and bias in the department of radiology: A pictorial essay**

**ELECTRONIC SUPPLEMENTARY MATERIAL**

**Supplementary Table 1** Summarized errors’ types categorization and related errors management strategies

| Errors’ types | | | | Related errors management strategies |  |
| --- | --- | --- | --- | --- | --- |
| Professional causes | Perceptual errors | Related to lesion size and density/signal | | Increased time for careful reading, bilateral contrast, and postprocessing methods such as maximum intensity projection (MIP), multiplanar reformation (MPR), and three-dimensional (3D) reconstruction |  |
|  |  | Related to the location/type of lesions | | Increasing time for comprehensive imaging reading and being more familiar with common blind areas, easily missed lesion types and the metastatic pattern of neoplastic diseases |  |
|  |  | Insufficiency of clinical information | | Improving hospital information systems to ensure adequate clinical and pathological information and increasing sensitivity of radiologists towards complications following surgery |  |
|  |  | Related to thinking bias | | Reading images [systematically](javascript:;), initiating a secondary search to continue looking for others after finding the first abnormality, and having a comprehensive knowledge of common diagnostic combinations |  |
|  |  | Limitations of equipment and post-processing software | | Increasing investment of time and effort in equipment development and perfection, thereby reducing diagnostic errors caused by equipment issues |  |
|  | Cognitive errors | Related to lack of professional knowledge | | Attaching importance to the training of professional knowledge by report writing training and continuing education for radiologists |  |
|  |  | Related to the prior examination and report | | Reading the images carefully and making a diagnosis before reviewing previous reports, revisiting and refining previous reports, and considering a second diagnosis |  |
|  |  | Related to clinical information | | Asking for the clinical history or physical examination in person |  |
|  |  | Related to thinking cognitive bias | Anchoring bias and confirmation bias | Reviewing and gathering all available evidence before the final diagnosis is made, especially evidence supporting a different opinion, and then considering a second diagnosis | |
|  |  |  | Availability bias | Using objective data of the disease incidence to correlate with the radiologist’s diagnostic rates and make a differential diagnosis | |
|  |  |  | Attribution bias | Realizing that initial clinical impressions can sometimes be wrong and reviewing the images before checking the clinical history | |
|  |  |  | Outcome bias and premature closure | Realizing the above cognitive biases that can influence our diagnosis and conducting a complete and accurate diagnosis with a correct attitude | |
| Non-  professional causes | Fatigue | | | Taking regular breaks to avoid fatigue |  |
|  | Inadequate attention of the readers | | | Moving and standing from time to time when reading images, minimizing interruptions in the diagnostic process caused by phone calls |  |
|  | Radiologists suffer neck and shoulder pain, back pain, carpal tunnel syndrome, eye strain, headache and other symptoms that may interfere with work | | | Using height-adjustable desks and ergonomic chairs, adjusting the brightness of indoor lighting and screens, and reducing ambient noise |  |
